# Temperature-induced modulation of stress-tolerant PGP genes bioprospected from *Bacillus* sp. IHBT-705 associated with saffron (*Crocus sativus)* rhizosphere: A natural -treasure trove of microbial biostimulants

**DOI:** 10.3389/fpls.2023.1141538

**Published:** 2023-02-27

**Authors:** Nilofer Ali, Mohit Kumar Swarnkar, Raj Veer, Priya Kaushal, Aparna Maitra Pati

**Affiliations:** ^1^ Biotechnology Division, CSIR-Institute of Himalayan Bioresource Technology, Palampur, India; ^2^ Academy of Scientific and Innovative Research (AcSIR), Ghaziabad, India; ^3^ Incubatee at Chief Minister Startup Scheme, Shimla, India

**Keywords:** saffron rhizosphere, stress tolerant PGP potential, long-read sequencing, gene expression, biostimulants for soil and plant health

## Abstract

There is a renewed interest in sustainable agriculture wherein novel plant growth-promoting rhizobacteria (PGPR) are being explored for developing efficient biostimulants. The key requirement of a microbe to qualify as a good candidate for developing a biostimulant is its intrinsic plant growth-promoting (PGP) characteristics. Though numerous studies have been conducted to assess the beneficial effects of PGPRs on plant growth under normal and stressed conditions but not much information is available on the characterization of intrinsic traits of PGPR under stress. Here, we focused on understanding how temperature stress impacts the functionality of key stress tolerant and PGP genes of *Bacillus* sp. IHBT-705 isolated from the rhizosphere of saffron (*Crocus sativus*). To undertake the study, *Bacillus* sp. IHBT-705 was grown under varied temperature regimes, their PGP traits were assessed from very low to very high-temperature range and the expression trend of targeted stress tolerant and PGP genes were analyzed. The results illustrated that *Bacillus* sp. IHBT-705 is a stress-tolerant PGPR as it survived and multiplied in temperatures ranging from 4°C-50°C, tolerated a wide pH range (5-11), withstood high salinity (8%) and osmolarity (10% PEG). The PGP traits varied under different temperature regimes indicating that temperature influences the functionality of PGP genes. This was further ascertained through whole genome sequencing followed by gene expression analyses wherein certain genes like *cspB, cspD, hslO, grpE*, *rimM, trpA, trpC, trpE, fhuC, fhuD*, *acrB5* were found to be temperature sensitive while, cold tolerant (*nhaX* and *cspC)*, heat tolerant *(htpX)* phosphate solubilization (*pstB1)*, siderophore production (*fhuB* and *fhuG*), and root colonization (*xerC1* and *xerC2*) were found to be highly versatile as they could express well both under low and high temperatures. Further, the biostimulant potential was checked through a pot study on rice (*Oryza sativa*), wherein the application of *Bacillus* sp. IHBT-705 improved the length of shoots, roots, and number of roots over control. Based on the genetic makeup, stress tolerance potential, retention of PGP traits under stress, and growth-promoting potential, *Bacillus* sp. IHBT-705 could be considered a good candidate for developing biostimulants.

## Introduction

PGPRs are now widely recognized for promoting sustainable agriculture (Gouda et al., 2018) for their ability to enhance plant productivity in multiple ways (Raza et al., 2016). PGPRs enable higher nutrient acquisition through the solubilization of phosphate (Ahemad and Khan, 2012), the production of siderophore for better uptake of iron (Beneduzi et al., 2012), the synthesis of plant growth regulators like indole acetic acid, the release of volatile organic compounds, the production of antifungal enzymes like chitinase, glucanase, and ACC-deaminase (1-Aminocyclopropane-1-carboxylate) and inducing resistance against pathogens (Choudhary et al., 2011; García-Fraile et al., 2015). Numerous studies have illustrated the beneficial application of PGPR under stress (Sapre et al., 2018; Ali and Khan, 2021). PGPRs improved yield, and tolerance against salinity stress in wheat (Lastochkina et al., 2017; Ansari et al., 2019), enhanced tolerance to drought in chickpeas (Vurukonda et al., 2016; Khan et al., 2019), and enhanced the growth of the tomato under cold stress (Subramanian et al., 2016). Under heat stress, thermotolerant PGPRs stimulated heat-responsive genes (*GmHSP*, *GmLAX3*, and *GmAKT2*) in soybean (Khan et al., 2020). Despite the multitude of positive traits, the commercial application of PGPR as a biostimulant experiences certain limitations, the most common being the variability of performance under field conditions (Timmusk et al., 2017; Glick, 2018). This is primarily due to the mode of selection process wherein PGP traits of microbes are assessed at a laboratory scale under highly controlled conditions, while field conditions are markedly different (Vasseur-Coronado et al., 2021). In fields, multiple factors interact, hence the PGP potentials of microbes are often lost or masked leading to underperformance (Backer et al., 2018; Mellidou and Karamanoli, 2022). Hence, the selection of the right microbe which does not lose its PGP traits under field conditions and various stresses is crucial (Amaya-Gómez et al., 2020). Unraveling the genetic makeup of PGPR can help in understanding its stress tolerance potential and predict its functionality as a biostimulant. The expression trend of genes will have a direct effect on their PGP potential and field performance (Bruto et al., 2014). Hence in this study, we analyzed the PGP traits of *Bacillus* sp. IHBT-705 under stressed conditions and carried out gene expression studies of key PGP genes over a wide range of temperatures to understand how temperature impacts their functionality.

*Bacillus* sp. IHBT-705 was isolated from the rhizosphere of saffron (*Crocus sativus*) cultivated in Patti village, Kangra District, Himachal Pradesh India since 2018. Among several PGPRs isolated from the region, *Bacillus* sp. IHBT-705 was found to highly stress tolerant and interestingly its key PGP traits like phosphate and potassium solubilizing potential, siderophore IAA and ACC deaminase, were evident over varied temperature regimes. Hence, the present study focused on assessing the stress tolerance capacity and analyzing the PGP trait of the bacteria, *Bacillus* sp. IHBT-705. We also wanted to understand the underlining functioning of key PGP genes in response to different temperature regimes i.e., very low (4°C) to very high (50°C). To elucidate the functional characteristics of PGP and stress-related genes, whole genome sequencing of *Bacillus* sp. IHBT-705 was carried out using a long-read sequencing system, PacBio RS II (Pacific Bioscience, USA). Genome sequencing followed by data mining lead to the identification of several key genes associated with stress tolerance and PGP traits. Further, the expression analyses of key PGP genes were studied in detail under varied temperature regimes. This study provided new insight into understanding the functionality of PGP-related genes of *Bacillus* sp. IHBT- 705 over a wide range of temperatures. For the first time, the study illustrated that certain PGP genes are highly sensitive to temperature while some genes are versatile and can function well over wide temperature regimes.

## Materials and methods

### Sampling and physiochemical properties of soil

Soil samples were collected from the rhizosphere of saffron (*Crocus sativus*) grown at Patti village, Palampur (HP), India (32.0708° North & 76.5415° East) (**Supplementary Figure S1**) from a depth of 8- 15cm. Five independent soil samples were collected from five different places in the field, wherein each sample consisted of four subsamples pooled together from the four sides of the saffron (*Crocus sativus*) rhizosphere from the same site. Physiochemical properties of soil i.e pH, electrical conductivity (EC), Distribution of Nitrogen (N), Phosphorus (P), Organic carbon (OC), Potassium (K), Iron (Fe), Manganese (Mn), Zinc (Zn), Calcium (Ca) and Copper (Cu) were analyzed. The pH of the soil and electrical conductivity were measured by immersing the electrodes into the soil: water-1:2.5 (w:v) extraction by using a calibrated pH meter (Thermo Scientific Eutech PC450, USA) and conductivity meter (Thermo Scientific Eutech Cyber Scan CON45, USA). Total N in the soil was determined by the micro-Kjeldahl method after digestion in concentrated sulfuric acid. The total P was determined by Bray’s method (Bray and Kurtz, 1945), whereas the OC was estimated as per the standard dichromate oxidation method (Nelson and Sommers, 1983). Available K, Fe, Mn, Zn, Ca, and Cu was determined by using a flame photometer (model BWB XP, BWB Technologies UK Ltd., UK) after Mehlich -3 extraction (Mehlich, 1984).

### Isolation of PGP bacteria

Soil samples were serially diluted (10^-8^), spread on Tryptone Soya Agar (TSA) media plates (Himedia, India) incubated in an incubator shaker (Innova 12444, New Brunswick Scientific, USA) at 28 ± 0.1°C for 48 hrs for the isolation of PGP bacteria. The morphology of isolated colonies was observed for shape, texture, color, margin, opacity, and elevation. Gram’s reaction was performed as per the method optimized by Vincent (Vincent, 1970). Pure colonies were preserved in glycerol stock (−80°C) till further use.

### Stress tolerance potential of *Bacillus* sp. IHBT-705

To check the temperature tolerance, *Bacillus* sp. IHBT-705 was grown in Tryptone Soya Broth (TSB) media (Himedia, India) at 180 rpm for 48 hrs in an incubator shaker at ambient temperature (28°C). After 48 hrs, the optical density of bacterial suspension was observed at 600nm and found to be 1 (OD-1) and 100 µl of this bacterial suspension was inoculated in 100 ml of TSB media and incubated at targeted temperatures i.e very low (4°C and15°C), low (20°C), optimum (28°C), high (42°C) and very high (50°C) in an incubator shaker. For pH tolerance, pH (4, 5, 6, 7, 8, 9, 10, 11, and 12) was adjusted in TSB media by 1N NaOH and 1M HCl. Thereafter 100 µl of 48 hrs old *Bacillus* sp. IHBT-705 suspension (OD-1) was inoculated in 100 ml of selected pH media and incubated at targeted temperatures in an incubator shaker (Getahun et al., 2020). For salinity tolerance, concentrations of sodium chloride (0%, 2%, 4%, 6%, 8%, 10%, 12%, and 15%) were varied in TSB media and 100 µl of 48 hrs old *Bacillus* sp. IHBT-705 suspension (OD-1) was inoculated in 100 ml of selected NaCl TSB media and incubated at targeted temperatures in an incubator shaker (Ramadoss et al., 2013). For osmotic tolerance, concentrations of polyethylene glycol (PEG-10,000MW) (0%, 5%, 10%, 15%, 20%, 25%, and 30%) were varied in TSB media. Thereafter, 100 µl of 48 hrs old *Bacillus* sp. IHBT-705 suspension (OD-1) was inoculated in 100 ml of selected PEG TSB media and incubated at targeted temperatures in an incubator shaker (Niu et al., 2018). The optical density of bacterial growth was measured with a microplate reader (Synergy™ LX Multi-Mode BioTek) and incubated in an incubator shaker (Innova 12444, New Brunswick Scientific, USA) at 180 for 48 hrs for all the abiotic stresses mentioned above at targeted all temperatures.

### PGP potential of *Bacillus* sp. IHBT-705

The initial screening and qualitative estimation of inorganic phosphate (P) solubilization of *Bacillus* sp. IHBT-705 was done on Pikovskaya’s (PVK) agar plates (Nautiyal, 1999). Qualitative estimation of potassium (K) solubilization assay was done on Aleksandrow agar media (Dhaked et al., 2017). The siderophore production was checked by Chrome Azurol S assay (CAS) (Schwyn and Neilands, 1987). The IAA production ability was evaluated by a colorimetric detection test (Loper, 1986). ACC deaminase assay was done according to the method proposed by Dworkin and Foster (Dworkin and Foster, 1958). All PGPR attributes were done at all targeted temperatures. The qualitative estimation of P, K solubilization and siderophore production was calculated by solubilization index (SI) =colony diameter + halo zone diameter/colony diameter (Paul and Sinha, 2013).

### Molecular identification based on 16S rRNA gene sequencing

For molecular identification, the genomic DNA of *Bacillus* sp. IHBT-705 was extracted by using the GenElute bacterial genomic DNA isolation kit (Sigma-Aldrich, USA) and the quality and quantity of genomic DNA were determined by gel electrophoresis (Bio-Rad, USA) and NanoDrop 2000 (Thermo Scientific, USA) and Qubit version 2.0 fluorometer (Invitrogen, USA) respectively. The 16S rRNA amplification was performed by polymerase chain reaction (PCR) using a genomic DNA template and universal primers 27F (5’AGA-GTT-TGA-TCC-TGG-CTC-AG3’) and 1492R (5’TAC-GGT-TAC-CTT-GTT-ACG-ACT3’) were used to a near-full length, approximately 1500 bp PCR product (Weisburg et al., 1991). For PCR amplification, 25µl standard reaction mixture containing: GoTaq Green Master Mix 1x, primer (forward and reverse primer,1µM each), and DNA template was prepared and cycled at 94°C for 2min for initial denaturation, followed by 35 cycles consisting of denaturation at 94°C for 30sec, annealing at 55°C for 30sec, and elongation at 72°C for 2min; a final 7min elongation step at 72°C. The amplified product was visualized on a 1% agarose gel under UV light for a positive amplification signal, and the PCR product was purified Exosap-IT solution (Applied Biosystem, USA) according to the manufacturer’s instructions. DNA ladder (1Kb, Qiagen, Netherlands) was used for size identification. Big Dye Terminator cycle sequencing kit v3.1 (Applied Biosystems, USA) was used according to the manufacturer’s instructions and its PCR product was purified by Montage sequencing reaction kit (Millipore USA). The purified PCR product was injected into a Genetic analyzer (Applied Biosystems, USA) for Sanger sequencing technology. Sequences obtained were submitted as a query to the BLASTn (https://www.ncbi.nlm.nih.gov/) search algorithm. Strain identification was done at the species level using EzTaxon (https://www.ezbiocloud.net/) and sequences of homologous strains were aligned using CLUSTALW (Larkin et al., 2007), and a phylogenetic tree was constructed with the MEGA version 11 (Tamura et al., 2021) (https://www.megasoftware.net/) using the neighbor-joining method with 1,000 bootstrap replicates (Saitou and Nei, 1987).

## Pot experiment

### Inoculation of *Bacillus* sp. IHBT-705 on rice (*Oryza sativa*)

To carry out the germination assay, seeds of Rice (HPR 2880 B/S) procured from CSKHPKV university Palampur Himachal Pradesh India, were surface sterilized (Nezarat and Gholami, 2009) and the experiment was set up in five replicates with two treatments i.e., treated (*Bacillus* sp. IHBT-705) and control (without *Bacillus* sp. IHBT-705). For the treated set, *Bacillus* sp. IHBT-705 was grown in TSB (Himedia, India) and incubated in an incubator shaker (Innova 12444, New Brunswick Scientific, USA) at 180 rpm for 24 hrs at 28°C, thereafter, centrifuged at 8000 rpm for 10 min. The centrifuged pellet was resuspended in autoclaved water for making OD-1 and used for further experiments. The effect of *Bacillus* sp. IHBT-705 on the growth of rice was studied by soaking rice seeds in the *Bacillus* sp. IHBT-705 suspension (OD-1) for 30 min. The seeds of control set were soaked in sterile distilled water. Each pot was filled with 2 kg autoclaved soil and fifty soaked seeds were planted, thereafter10 ml of *Bacillus* sp. IHBT-705 suspension was added in treated set. Seeds treated with sterile distilled water without bacterial suspension were taken as control. The treatments were arranged in a completely randomized design (CRD) with five replicates and placed in a glass house. After 60 days of sowing, ten plants were randomly uprooted and washed under running water, and root/shoot lengths and numbers of roots were measured.

### Complete genome sequencing and annotation of *Bacillus* sp. IHBT-705

Complete genome sequencing was carried out to understand the genomic complexity of *Bacillus* sp. IHBT-705. The genomic DNA of *Bacillus* sp. IHBT-705 was isolated as mentioned above and ~10µg of high-quality intact genomic DNA was used for long reads library preparation using a PacBio SMRTbell template preparation kit with 20Kb insert size (Pacific Biosciences, USA) version 1.0 as per manufacturer’s instructions. Briefly, intact gDNA was fragmented using g-tubes (Covaris, Inc. USA) and the quality of the sheared DNA was checked using gel electrophoresis (**Supplementary Figure S2A**). Followed by end-repairing, adaptor ligation, and purification of library molecules using Bluepippin size-selection system (Sage Science, USA) called SMRTbell template (**Supplementary Figure S2B**). The QC of the library was checked with Bioanalyzer DNA 12000 chip (Agilent Technologies, USA) and Qubit Fluorometer v3.0 (Invitrogen, USA) as shown in (**Supplementary Figure S2C**). Further, genome sequencing of *Bacillus* sp. IHBT-705 was performed on the PacBio RS II system with polymerase P6-C4 sequencing chemistry. The annotation was performed using Rapid Annotation Using Subsystems Technology (RAST) (Aziz et al., 2008). Denovo assembly of sequenced sub-reads was carried out using HGAP (Hierarchical Genome Assembly Process) (**Supplementary Figure S3**) protocol version 3.0 in SMRT analysis version 2.2.0 (Pacific Biosciences, USA) and produced a complete circular genome sequence with high coverage.

### Whole-genome alignment with closest strain

For comparative genome analysis reference genome sequence of *Bacillus altitudinis* ASM97268 (Top hit in 16S rRNA Sequencing) was downloaded from the NCBI (Accession number NZ_CP011150.1), and imported into the CLCBio genomics workbench (Qiagen, Netherlands). The complete genome sequence of *Bacillus* sp. IHBT-705 (CP074101) was used against *Bacillus altitudinis* (ASM97268) for whole-genome alignment. The alignment was performed using CLC Genomics Workbench (v21.0.3) whole-genome alignment tool with default parameters (minimum initial seed length 15bp and minimum alignment block length (100bp). This tool primarily works by identifying seeds i.e., short stretches of nucleotide sequence that are shared between multiple genomes but not present multiple times in the same genome. These seeds are extended using the HOXD scoring matrix (Chiaromonte et al., 2002) until the local alignment score drops below a fixed threshold.

### Genome-based average nucleotide identification with closest strain

The genomic similarity between the *Bacillus* sp. IHBT-705 and the closest strain *B*. *altitudinis* ASM97268 from the NCBI database were calculated using the Orthologous Average Nucleotide Identity algorithm (OrthoANIu) (https://www.ezbiocloud.net).

### Expression analysis of stress-tolerant and PGP genes

Genes responsible for stress (cold and heat), pH tolerance, and PGP attributes such as Phosphate solubilization, IAA production, siderophore production, ACC deaminase activity, and root colonization were selected based on the available literature (**Table 1**) and screened from the whole genome annotation of selected PGP and stress-tolerant bacteria by BLAST to GO (Conesa et al., 2005) for expression studies. Primers for stress-tolerant and PGP genes were designed by using the Primer Express software version 3 (Applied Biosystem USA) as shown in (**Supplementary Table S1**).

**Table 1:** List of stress-tolerant and PGP genes selected from the whole genome of *Bacillus* sp. IHBT-705 for expression studies.

Genes	Reported Attributes	Ref.
*nhaX* *cspD* *cspB* *cspC*	Cold Shock Proteins	(Gupta et al., 2014; Kishor PB, 2019; Lalaouna et al., 2019)
*dnaJ* *dnaK* *hslO* *htpX* *grpE*	Heat Shock Genes	(Gupta et al., 2014; Suarez et al., 2019)
*trpA* *trpB* *trpD* *trpC* *trpE*	IAA Biosynthesis.	(Gupta et al., 2014; Dahmani et al., 2020; Zaid et al., 2022).
*fhuC* *fhuD* *fhuB* *fhuG* *acrB5*	Siderophore Production	(Gupta et al., 2014; Suarez et al., 2019)
*nhaK*	pH tolerance	(Noori et al., 2021)
*pstB1*	Phosphate Solubilization	(Suarez et al., 2019)
*rimM*	ACC Deaminase Activity	(Gupta et al., 2014)
*xerC1* *xerC2*	Rhizosphere Colonizer	(Shen et al., 2013; Eida et al., 2020)

### RNA extraction, cDNA synthesis, and qRT-PCR analyses

*Bacillus* sp. IHBT-705 was grown in TSB media (Himedia India) at all targeted temperatures at 180 rpm for 24 hrs in an incubator shaker and after 24 hrs total RNA was isolated separately from *Bacillus* sp. IHBT-705 suspension (OD-1) by using RNeasy Mini Kit (Qiagen, Netherlands). The quality of RNA was checked by using RNA nanochip on a bioanalyzer (Agilent Technologies, USA). Further, the RNA quality and quantity were verified by agarose gel electrophoresis (Bio-Rad, USA) followed by ND-1000 NanoDrop (Thermo Scientific, USA) respectively. Total RNA was reverse transcribed into cDNA using a verso cDNA synthesis kit (Thermo Fisher Scientific, USA) according to the manufacturer’s instructions. Briefly, the reaction was prepared in 20µl volume with random hexamer primer conducted at 42˚C for 60min and 95˚C for 2min for heat denaturation of enzymes. Prepared cDNA was confirmed by PCR amplification using 16S primer pair followed by agarose gel electrophoresis (Bio-Rad, USA) (Cárdenas Espinosa et al., 2021). Real-time qRT-PCR was performed with Syber Green PCR Master Mix (Thermo Fisher Scientific, USA) and conducted on Step One Plus 2.0 Real-Time PCR System (Applied Biosystems, USA). The reaction mixture included 2μl of diluted cDNA (1:10), 0.5μl each primer, and SYBR green 5μl. The final reaction volume of 10μl was adjusted with nuclease-free water. The following PCR condition was used: 95˚C for 7min, 40 cycles of 95˚C for 15sec, and 60˚C for 30sec. The melting temperature-determining dissociation step was run at 95˚C for 15sec and 60˚C for 1min and 95˚C for 15sec at the end of the amplification. All the reactions were carried out in triplicate for each cDNA sample (Jian et al., 2020).

## Statistical analysis

Relative gene expression calculations of stress-tolerant and PGP genes were calculated according to the comparative critical threshold (2ΔΔCT) method (Livak and Schmittgen, 2001). The expression of two housekeeping genes (*16S*, *secA*) was analyzed for stress-tolerant and PGP gene primers. At all targeted temperatures, the expression of the *16S* gene was highly stable, and similar results were obtained by using it as a normalization gene at 28˚C (optimum temperature for gene expression) and comparison of Ct-values further revealed that expression of *16S* reference gene was stably expressed with constant Ct values (**Supplementary Figure S4A, B**). Reference gene *secA* was not stably expressed in the targeted temperatures. Hence, only the 16S reference gene was considered as the internal reference. All experiments were performed in triplicates for gene expression and in five replicates for pot experiments. All data (Significance in fold-change and Pot experiment) was analyzed by using one-way ANOVA followed by Duncan multiple range test (DMRT) (p-value = 0.05) (https://ccari.icar.gov.in/wasp/index.php).

## Results

### Physicochemical properties of soil

We wanted to understand the nature of soil being the habitat of *Bacillus* sp. IHBT-705, hence the soil characteristics (pH, EC, available N2, P, OC, K, Fe Mn, Zn, Ca, and Cu) were analyzed (**Table 2**).

**Table 2:** Physiochemical properties of rhizosphere soil. Values represented are mean ± standard error of three replicates.

pH	EC	N_2_	P	K	OC	Zn	Fe	Ca	Cu	Mn
	(dS m^-1^)	Kg/ha			%	ppm				
4.8 ± 0.02	131 ± 0.04	294.57 ± 0.02	19.29 ± 0.25	22.2 ± 0.02	2.81 ± 0.02	15 ± 0.02	453.23 ± 0.07	5.26 ± 0.24	12.73 ± 0.02	8.4 ± 0.03

### Morphological characterization of *Bacillus* sp. IHBT-705

Colonies of *Bacillus* sp. IHBT-705 were off-white, translucent, elevated, and round with a regular margin. Gram staining illustrated it to be rod-shaped gram-positive bacteria.

### Stress tolerance potential of *Bacillus* sp. IHBT-705

**Figure 1:**
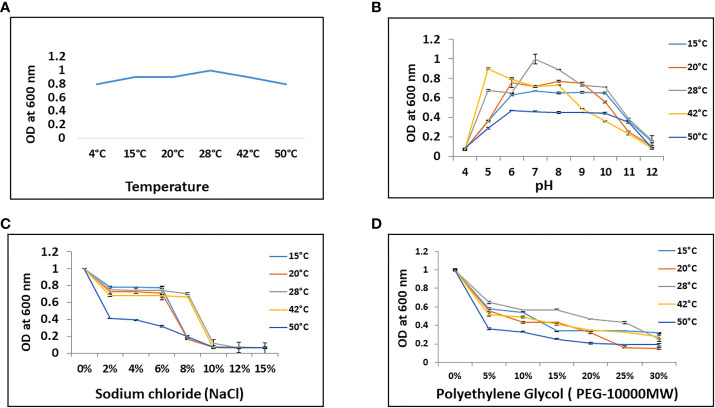
Stress tolerance potential of *Bacillus* sp. IHBT-705. **(A)** Thermotolerance potential. **(B)** pH tolerance. **(C)** Salt (NaCl) tolerance. **(D)** Osmotic (PEG 10,000MW) tolerance. All the experiments were done on targeted temperatures and Error bars represent the standard error of three replicates.

*Bacillus* sp. IHBT-705 exhibited growth on temperatures ranging from 4°C- 50°C on TSA as well as in TSB medium. However, 28°C was found to be the optimum temperature for growth and multiplication (**Figure 1A**). We wanted to understand the nature of *Bacillus* sp. IHBT-705 particularly under a stressed environment, so we subjected it to different stresses like temperature, pH, salinity, and osmoticum. *Bacillus* sp. IHBT-705 exhibited thermotolerance on temperatures ranging from 4°C- 50°C (**Figure 1A**) and could multiply at pH ranging from 5 to 11 at all the targeted temperatures indicating that it can tolerate and propagate both in acidic as well as the alkaline environment (**Figure 1B**). The salinity (NaCl) tolerance level of *Bacillus* sp. IHBT-705 was up to 6% at all the targeted temperatures and increased up to 8% at 28°C and 42°C (**Figure 1C**). *Bacillus* sp. IHBT-705 exhibited relatively slow growth in medium supplemented with PEG-10,000MW (10% onwards) from 15°C-42°C and almost no growth at 50°C (**Figure 1D**). All these stress assessment results indicate *Bacillus* sp. IHBT-705 to be a stress-tolerant PGP.

### PGP potential of *Bacillus* sp. IHBT-705

Though *Bacillus* sp. IHBT-705 was found to be a stress-tolerant PGPR, but it was essential to check if it retained its growth-promoting attributes under stress or not so that it can serve as an effective biostimulant. Hence, we checked the key PGP traits under all the targeted temperatures. The P solubilization and siderophore production of *Bacillus* sp. IHBT-705 was confirmed by a clear zone around the colonies on PKV and blue CAS agar media at all targeted temperatures however, the potential varied. K solubilization by *Bacillus* sp. IHBT-705 was confirmed by a clear zone around the colonies on Aleksandrow medium at all targeted temperatures except 4°C, IAA production by the pink color formation in the salkowaski reagent at all targeted temperatures except 4°C and ACC deaminase activity was confirmed by the growth of *Bacillus* sp. IHBT-705 on DF salt minimal medium at all targeted temperatures except 4°C (**Table 3**).

**Table 3:** PGP Potential of *Bacillus* sp. IHBT-705. Values represented are the mean of three replicates.

PGPR Strain	Targeted temperatures	Phosphate Solubilization (Zone in mm)	Potassium Solubilization (Zone in mm)	Siderophore production (Zone in mm)	IAA Synthesis	ACC Deaminase Activity
***Bacillus* sp. IHBT-705**	4°C	7	–	8	–	–
	15°C	10	7	12	+	+
	20°C	10	7	12	+	+
	28°C	12	10	15	++	+
	42°C	12	8	15	++	+
	50°C	10	6	12	+	+

+ indicates moderate activity, ++ indicates high activity, - indicates absence of activity.

### Molecular identification based on 16S rRNA gene sequencing

**Figure 2:**
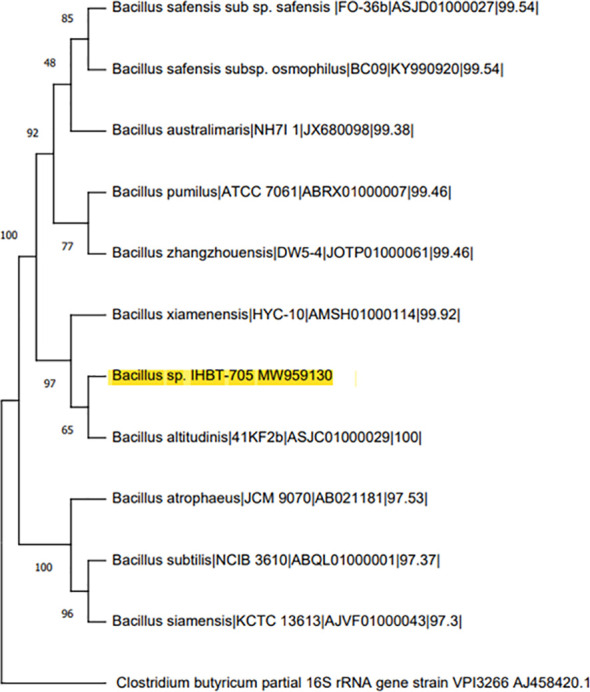
Phylogenetic relationships of 16S sequence of *Bacillus* sp. IHBT-705 and its homologs to the other species within the genus *Bacillus* and *Clostridium butyricum* as an outgroup.

Phylogenetic analysis of *Bacillus* sp. IHBT-705 based on 16S rRNA sequence showed a close evolutionary relationship of 99.46% similarity with *Bacillus altitudinis* (ASJC01000029) as per the closest neighbor-joining method (**Figure 2**). The16S rRNA nucleotide sequence of *Bacillus* sp. IHBT-705 has been submitted to the NCBI gen bank under accession number MW959130 and its pure culture was deposited under the accession number MTCC25416 at the Microbial Culture Collection (MCC) at IMTECH, Chandigarh, India.

### Effect of *Bacillus* sp. IHBT-705 on rice

**Figure 3:**
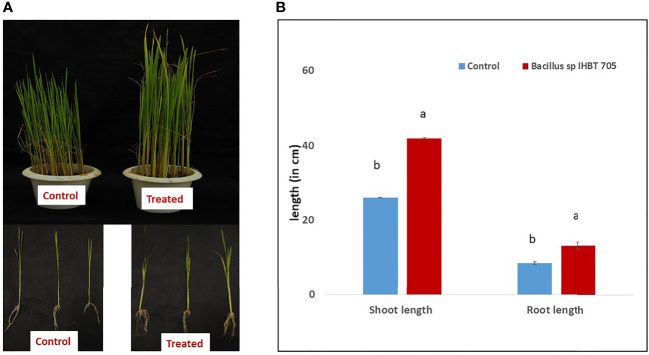
Effect of *Bacillus* sp. IHBT-705 on Rice **(A)** growth of rice seedlings under-treated and control conditions **(B)** shoot and root length. Different letters indicate significant differences among the treated and control treatments at p = 0.05. Data (mean ± SE) are averages of five replicates (n=5).

Through *in vitro* assay the *Bacillus* sp. IHBT-705 was found to exhibit promising plant growth potential this was further investigated through a pot study. *Bacillus* sp. IHBT-705 inoculation positively affected the growth of rice seedlings (**Figure 3A**). The treatment of rice with *Bacillus* sp. IHBT-705 significantly increased the length of shoots (42 cm ±0.1) as compared to the control treatment (28 cm ±0.3). Similarly, the length of roots was higher in *Bacillus* sp. IHBT-705 treated plants (13.08cm ±0.20) as compared to the control treatment of (8.48 ± 0.40; **Figure 3B**). Importantly, the numbers of total roots were higher in treated plants (85.58 ± 0.91) as compared to the control treatment (65.2 ± 1.78).

### Complete genome sequencing of *Bacillus* sp. IHBT-705

**Figure 4:**
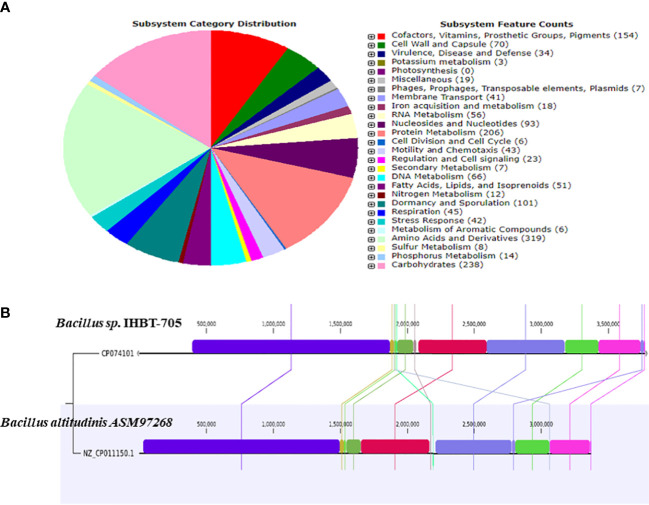
Genome annotation of *Bacillus* sp. IHBT-705. **(A)** Genome annotation of *Bacillus* sp. IHBT-705 using RAST (Rapid Annotations using Subsystems Technology). **(B)** Whole genome alignment of *Bacillus* sp. IHBT-705 (CP074101.1) with closest species reference genome *B. altitudinis* (NZ_CP011150.1).

The complete genome and plasmid sequences of *Bacillus* sp. IHBT-705 genome sequences were deposited at DDBJ/EMBL/NCBI GenBank under the accession numbers CP074101 under BioProject PRJNA725988 and BioSample SAMN18917214. Obtained a total of 312,490,430 bases and 39,800 reads (N50 Read length 10,827 bases and mean read length 7,851 bases). Out of which 36,038 reads (91%) were mapped and generated a complete assembly of 3,773,439 bases with 67.91X coverage and 41.3% GC content. Rapid Annotations using the Subsystems Technology (RAST) server predicted 4,015 genes for protein-coding (CDSs), 106 genes for RNAs, and 327 RAST subsystem categories were functionally assigned through the predicted genes as shown (**Figure 4A**). Out of 1682 features, 319 were found in amino acids and derivatives, 238 in carbohydrates and 206 in protein metabolism. The comparison of the *Bacillus* sp. IHBT-705 genome sequence with *Bacillus altitudinis* showed a close alignment using the whole-genome alignment tool as CLCBio Genomics Workbench (v21.0.3) with default parameters (minimum initial seed length 15bp and minimum alignment block length 100bp). The alignment was found to be matched at 5403 blocks (**Figure 4B**).

### Genome-based average nucleotide identification of *Bacillus* sp. IHBT-705 with closest strain *B. altitudinis* ASM97268

The draft genome length of *B. altitudinis* ASM 97268 was ~3.74 Mbp, while the genome size of *Bacillus* sp. IHBT-705 was ~3.77 Mbp. Close to 98.57% of total orthologous clusters were shared among two genomes.

### Expression analysis of stress-tolerant and PGP genes of *Bacillus* sp. IHBT-705 under selected temperatures

Having observed that *Bacillus* sp. IHBT-705 retains its major PGP traits under temperature stress, we wanted to understand the functionality of genes responsible for these traits. Not much information is available on the response of key PGP genes under varying temperature regimes, hence we carried out expression analysis of targeted genes to observe their modulation with the rise and fall of temperature. The observation of the study can pave the way to predict the efficiency of *Bacillus* sp. IHBT-705 as a biostimulant for plants growing in areas exposed to low and high temperatures. A total of twenty-four genes, governing stress (cold and heat), pH tolerance, and PGP attributes (IAA production, siderophore production, ACC deaminase activity, phosphate solubilization, and root colonization) were targeted based on the information available through literature (**Table 1**) for expression analysis studies.

**Figure 5:**
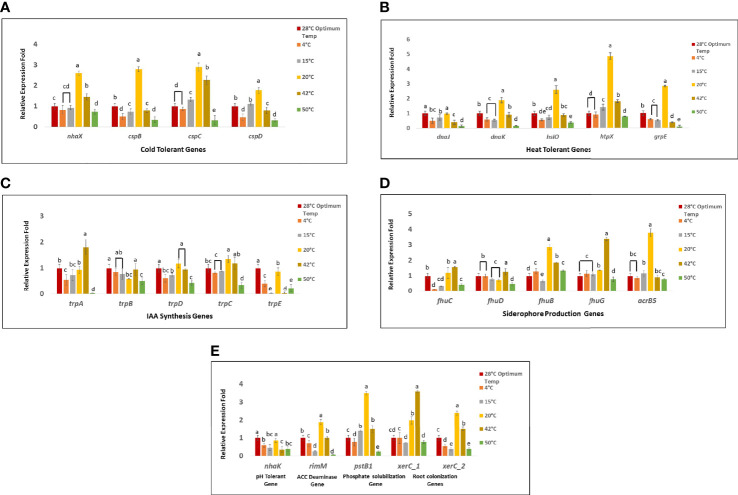
The relative expression of stress-related and PGP genes **(A)** Cold tolerant genes **(B)** Heat tolerant genes **(C)** Genes involved in IAA biosynthesis **(D)** Genes related to Siderophore-production **(E)** pH tolerant gene, ACC deaminase gene, phosphate solubilization and genes for root colonization. The results were represented as the mean of three replicates (n=3). Different letters indicate significant differences in gene expression among all targeted temperatures (P = 0.05).

Four genes *nhaX, cspB, cspC*, and *cspD* were targeted to unravel the cold tolerance capability of *Bacillus* sp. IHBT-705. Out of four cold tolerant genes, *cspC* was found to be versatile as it upregulated at 15°C, 20°C, and 42°C. Gene *nhaX* gene upregulated at 20°C and 42°C. Gene *cspB* and *cspD* both upregulated only at 20°C. Gene *cspB* downregulated at 4°C,15°C, and 42°C while gene *cspD* downregulated at 4°C and 42°C. All these four cold tolerant genes downregulated at 50°C (**Figure 5A**). Out of the five heat-tolerant genes (*dnaJ, dnaK, hslO, htpX*, and *grpE*), gene *dnaJ* did not upregulat at any targeted temperature while it stably expressed at 20°C and downregulated at all the other targeted temperatures. Both genes *dnaK*, and *hslO* upregulated at 20°C, stably expressed at 42°C and downregulated at 4°C, 15°C, and 50°C. While gene *htpX* upregulated at 15°C, 20°C, and 42°C, stably expressed at 4°C, and 50°C. Gene *grpE* upregulated only at 20°C and downregulated at all the other temperatures (**Figure 5B**). Out of five IAA synthesis genes *trpA, trpB, trpD, trpC*, and *trpE*, gene *trpA* upregulated at 42°C, stably expressed at 15°C and 20°C and downregulated at 4°C, and 50°C. Gene *trpB* stably expressed at 4°C, 15°C, and 42°C and downregulated at 20°C and 50°C. Gene *trpD* stably expressed at 20°C and 42°C and downregulated at 4°C, 15°C, and 50°C. Gene *trpC*, and *trpE* both upregulated at 20°C. Gene *trpC* expressed stably at 4°C, 15°C, 42°C and downregulated at 50°C. Gene *trpE* downregulated at all the targeted temperature except 20°C (**Figure 5C**). Genes *fhuB, fhuC, fhuD, fhuG*, and *acrB5* targeted for the siderophore production potential and out of these five genes, Genes *fhuC* and *fhuD* upregulated at 42°C, *fhuC* expressed stably at 20°C and *fhuD* at 4°C, both these genes downregulated on remaining other targeted temperatures. Gene *fhuB* upregulated at 4°C, 20°C 42°C and 50°C and downregulated at 15°C, while *fhuG* upregulated at 20°C and 42°C and stably expressed at 4°C and 15°C and downregulated at 50°C. Gene *acrB5* upregulated at 20°C and stably expressed at 4°C, 15°C, 42°C, and 50°C indicating retention of siderophore-producing activity at different temperature ranges (**Figure 5D**). Gene *nhaK* was targeted for pH tolerance and it stably expressed only at 20°C and downregulated at all the other targeted temperatures. Gene *rimM* gene targeted for ACC deaminase activity and it upregulated at only 20°C, stably expressed at 42°C and downregulated at all the other targeted temperatures. Gene *pstB1* targeted for the phosphate solubilizing potential and it upregulated at 15°C, 20°C, and 42°C and downregulated at 4°C and 50°C. Both the genes for root colonizations *xerC1* and *xerC2* upregulated at 20°C and 42°C, *xerC1* expressed stably and *xerC2* were downregulated at 4°C, 15°C and 50°C (**Figure 5E**).

## Discussion

Several species of *Bacillus* are well known for their plant growth-promoting attributes and during the present study plant growth-promoting potentials of *Bacillus* sp. IHBT-705 was unraveled through *in vitro* analyses, pot study, and gene expression studies. The bacteria exhibited thermo-tolerance, could multiply in the acidic and alkaline range and could tolerate salt and high PEG concentration indicating that it is a good stress-tolerant PGPR.

Upon whole genome analysis, several key genes like *cspB, cspC, cspD, nhaX*, and *htpX* were identified. These genes are known to confer cold stress tolerance in microbes (Kishor PB, 2019; Lalaouna et al., 2019; Suarez et al., 2019), hence it is likely that these genes could have conferred stress tolerance capability to *Bacillus* sp. IHBT-705. This gene expression study revealed that *nhaX* upregulated at low as well as high temperatures indicating that it could be responsible for imparting heat and cold tolerance and makes the bacterial strain tolerant to both cold and heat stress. Cold shock proteins (*csp*) family are known for their stress tolerance potential and found to be constitutively expressed during stress conditions in bacteria and even transgenic plants overexpressing bacterial *csp* exhibited tolerance to cold, salt, and drought stresses (Gupta et al., 2014; Kishor PB, 2019). In our study gene *cspC* overexpressed at high as well as low temperature suggesting that it may also impart heat and cold tolerance. Hence, presence of *cspC* gene makes *Bacillus* sp. IHBT-705 a valuable microbe from stress tolerance perspective. However, *cspB* and *cspD* exhibited a narrow range of functionality as both these genes upregulated only at low temperature.

Literature suggests that genes *dnaJ, dnaK, HslO, htpX*, and *grpE* are responsible for heat tolerance (Gupta et al., 2014; Suarez et al., 2019). However, we observed that genes *dnaK grpE, hslO* upregulated only at low temperature but not at high temperature, hence these genes were found to exhibit a narrow range of functionality. The gene *htpX* upregulated at low as well as high temperatures indicating its broad range of functionality and could have imparted stress tolerance ability, hence this gene appears to be crucial in conferring thermo-tolerance to *Bacillus* sp. IHBT-705. As the bacteria could tolerate up to 50°C, but none of the targeted gene upregulated at very high temperature further investigations by targeting other heat-tolerant genes are required to understand its extreme temperature tolerance capability.

The colorimetric tests illustrated that *Bacillus* sp. IHBT-705 efficiently produces IAA both at low as well as at high temperatures. Earlier studies indicate that *trp* genes (*trpA, trpB, trpD*, *trpC, trpE*) are involved in multiple biological processes including IAA biosynthesis (Gupta et al., 2014; Dahmani et al., 2020; Zaid et al., 2022). Interestingly, the present gene expression study revealed that specific temperature triggers the upregulation of specific sets of IAA-producing genes. Gene *trpA* was upregulated at high while *trpC* and *trpE* were upregulated at low temperature. The expression of *trpB* largely remained unaltered at both low and high temperatures, hence it could be considered a versatile gene with the ability to function over a broad temperature range with optimum efficacy. The role of microbial IAA in shaping root architecture ultimately benefitting plant productivity has been experimentally proven (Kazan, 2013; Sukumar et al., 2013; Grover et al., 2021), hence IAA is considered an important plant growth-promoting attribute, and in this respect, the complementary functioning of IAA-producing genes at both low and high temperature possibly enable retention of IAA producing capability of *Bacillus* sp. IHBT-705 under temperature stress which is a beneficial attribute.

Siderophore-mediated iron uptake in plants is a major benefit imparted by PGPRs (Braud et al., 2006; Mandal and Kotasthane, 2014). Siderophores are grouped into three principal categories based on their functional groups which are hydroxamates, catecholate, and carboxylates (Suarez et al., 2019; Kramer et al., 2020). Earlier studies revealed that the iron (III)-hydroxamate ABC transporter cluster *fhuCDB* and the ferric hydroxamate uptake gene *fhuA* are responsible for the transport of ferrichrome and other Fe3+ hydroxamate compounds (Gupta et al., 2014; Paul and Lade, 2014), hence in our study *fhuCDBG* and *acrB5* genes associated with the production of siderophore were targeted for expression analysis studies. Among these targeted genes, *fhuB* was found to be highly versatile as it upregulated from very low to very high temperatures. Similarly, *fhuG* upregulated from low to high temperature while *acrB5* upregulated at very low temperature, hence functionality of gene *fhuB* is a positive attribute of *Bacillus* sp. IHBT-705. Compared to IAA-producing genes, siderophore-related genes exhibited better expression at both low and high temperatures. The colorimetric study also illustrated that siderophore producing potential of *Bacillus* sp. IHBT-705 was active from very low to high temperature which is highly desirable trait from a biostimulant perspective.

The gene *nhaK* is known for imparting tolerance to pH (Noori et al., 2021), in this study gene *nhaK* was downregulated at all targeted temperatures thereby indicating a narrow range of functionality with respect to temperature. PGPRs produce extracellular polymeric substances and synthesize 1-aminocyclo-propane-1-carboxylate (ACC) deaminase enzyme or its homologue D-cysteine desulfhydrase, these enzymes lower ethylene accumulation in stressed plants (Sashidhar and Podile, 2010; Wagh et al., 2014) and enable plants to become stress tolerant (Paul and Lade, 2014). Gene *rimM* responsible for ACC deaminase activity (Gupta et al., 2014), *Bacillus* sp. IHBT-705 is an ACC- deaminase-producing strain consisting gene *rimM* and thus can act as a stress buster also. The expression analysis of *rimM* exhibited upregulation at low as well as a high temperature suggesting that *Bacillus* sp. IHBT-705 can function efficiently under temperature stress.

Qualitative assay phosphate solubilization potential of *Bacillus* sp. IHBT-705 was observed from very low to very high temperature which were further illustrated through gene expression study. The literature (Hudek et al., 2016; Suarez et al., 2019) suggests that *pstB1* plays a functional role in phosphate uptake. In our study gene *pstB1* upregulated from very low to high temperature, indicating that this gene is versatile and remains active both under low and high temperatures. This attribute is significant from a biostimulant perspective.

**Figure 6:**
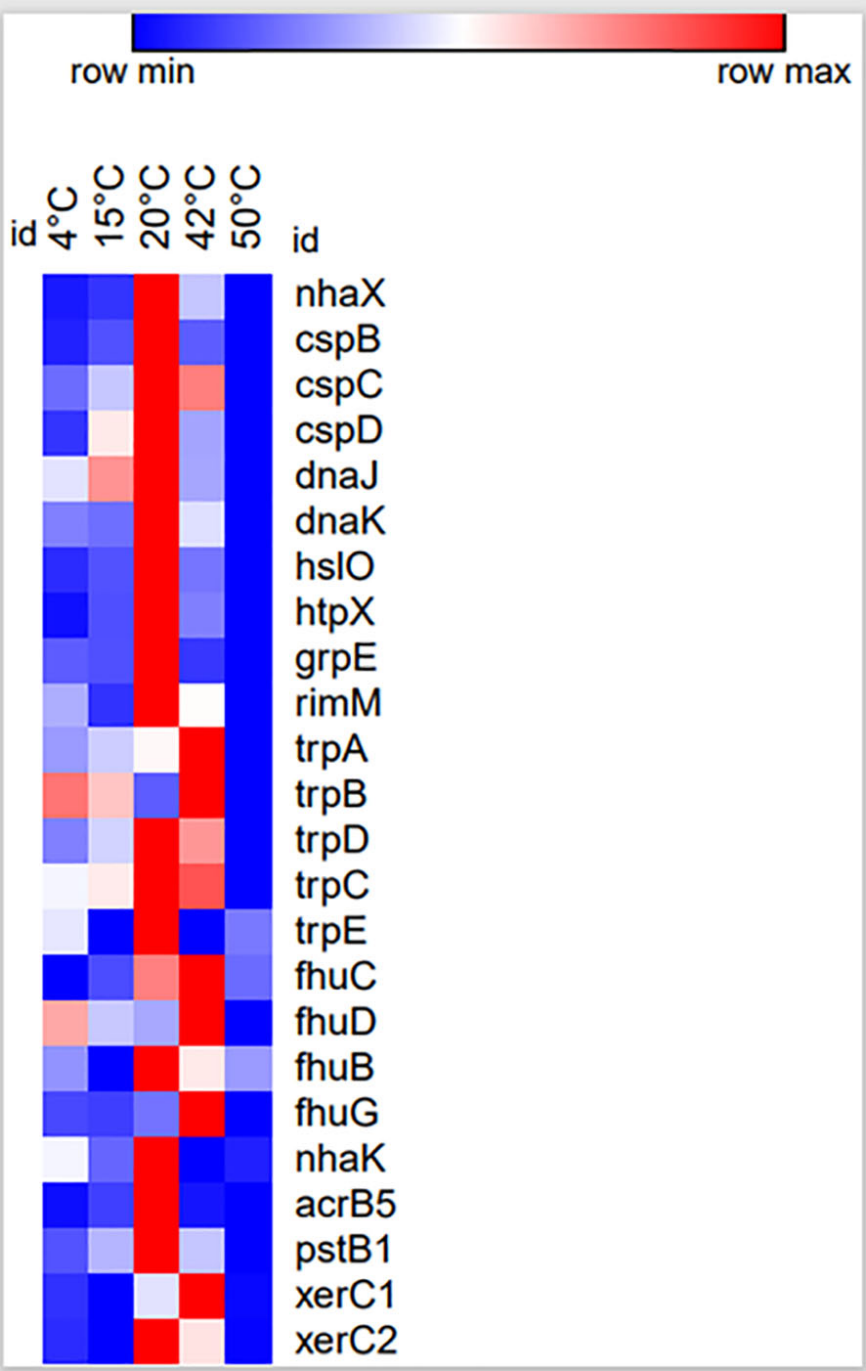
Heat map of twenty-four stress tolerant and PGP genes expression on targeted temperatures.

The ability to colonize the rhizosphere is an important attribute of PGPRs and genes *xerC1* and *xerC2* have been found to code site-specific recombinase which facilitates efficient colonization (Shen et al., 2013; Eida et al., 2020), hence these two genes were targeted. The expression analysis revealed overexpression of *xerC1* and *xerC2* at low and high temperature indicating that *Bacillus* sp. IHBT-705 could be a good colonizer both low and high temperatures. Apart from 28°C which is the optimum temperature for the growth of *Bacillus* sp. IHBT-705, among the targeted genes, the maximum number of genes were upregulated at 20°C and 42°C indicating its PGP potential at both low and high-temperature (**Figure 6**). Our study suggests that owing to its high-stress tolerance capability, *Bacillus* sp. IHBT-705 could be used as a potential biostimulant for crops growing in cooler mountainous regions as well as in warmer plains.

## Conclusion

Recent years have witnessed an upsurge in microbial-based biostimulant. Several studies on PGP traits illustrate that the intrinsic characteristics of microbes are critical to their biostimulant potential. In this regard, physiological, molecular, and gene expression study of *Bacillus* sp. IHBT-705 revealed that it could survive both under very low to very high temperatures, low and high pH, and withstand salinity and osmolarity, suggesting that it is a highly stress-tolerant PGPR. Whole genome sequencing revealed the presence of several genes responsible for imparting stress tolerance and multiple genes for a single PGP trait. Furthermore, expression analysis of key PGP genes revealed that temperature is a critical factor governing the upregulation/downregulation of specific PGP genes hence temperature strongly regulates the functionality of PGP traits. As a PGPR, *Bacillus* sp. IHBT-705 has unique genetic makeup with multiple genes for a specific PGP trait wherein the downregulation of one gene is compensated by the upregulation of complementary genes, making *Bacillus* sp. IHBT-705 a good candidate for developing biostimulant(s) for agriculture in hilly terrain, warmer plains, and land affected by sodic soils. The genes *nhaX, cspC, htpX, fhuB, fhuG, pstB1*, *xerC1, and xerC2* were found to be versatile genes owing to their expression both under low and high temperatures while *cspB, cspD, hslO, grpE*, *rimM, trpA, trpC, trpE, fhuC, fhuD*, *acrB5* were found to be temperature sensitive. The presence of stress-tolerant genes may serve as marker genes for selecting other PGPRs as stress tolerant PGPRs for developing biostimulants (s) suitable for agriculture in different terrains.

## Data availability statement

The datasets presented in this study can be found in online repositories. The names of the repository/repositories and accession number(s) can be found in the article/**Supplementary Material**.

## Author contributions

NA performed the experimentation, data analysis, and drafted the manuscript. MS contributed to experimentation and data analysis. RV helped in experimentation. PK edited and proofread the manuscript. AP conceptualized, guided, and structured the manuscript. All authors contributed to the article and approved the submitted version.

## Supplementary material

Supplementary Figure 1 Map of soil sampling site for isolation of PGPR.

Supplementary Figure 2 Library validation of gDNA for whole genome sequencing of *Bacillus* sp. IHBT-705. **(A)** Gel profile of isolated high-quality gDNA of *Bacillus* sp. IHBT-705 with 1Kb ladder. **(B)** Bioanalyzer profile of *Bacillus* sp. IHBT-705 SMRTbell templates for sequencing with ladder. **(C)** Bioanalyzer profile of prepared library size (~9.8 Kb) of *Bacillus* sp. IHBT-705.

Supplementary Figure 3 Genome assembly features of PacBio RS II data using HGAP v3.0. **(A)** Polymerase read length distribution of post-filter reads. **(B)** Polymerase read quality distribution of post-filter reads. **(C)** Subread filtering of the whole genome of *Bacillus* sp. IHBT-705 sequencing reads. **(D)** Coverage across a reference of single contig assembly (with an average coverage of 69X).

Supplementary Figure 4 Expressions analysis of targeted stress tolerant and PGP genes by qRT-PCR. **(A)** Amplification plots **(B)** Melt curves of targeted genes.

## References

[B1] Ahemad M. Khan M. S. (2012). Evaluation of plant-growth-promoting activities of rhizobacterium pseudomonas putida under herbicide stress. Ann. Microbiol. 62, 1531–1540. doi: 10.1007/s13213-011-0407-2

[B2] Ali S. Khan N. (2021). Delineation of mechanistic approaches employed by plant growth promoting microorganisms for improving drought stress tolerance in plants. Microbiol. Res. 249, 126771. doi: 10.1016/j.micres.2021.126771 33930840

[B3] Amaya-Gómez C. V. Porcel M. Mesa-Garriga L. Gómez-Álvarez M. I. (2020). A framework for the selection of plant growth-promoting rhizobacteria based on bacterial competence mechanisms. Appl. Environ. Microbiol. 86, e00760-20. doi: 10.1128/AEM.00760-20 32358015 PMC7357491

[B4] Ansari F. A. Ahmad I. Pichtel J. (2019). Growth stimulation and alleviation of salinity stress to wheat by the biofilm forming bacillus pumilus strain FAB10. Appl. Soil Ecol. 143, 45–54. doi: 10.1016/j.apsoil.2019.05.023

[B5] Aziz R. K. Bartels D. Best A. A. DeJongh M. Disz T. Edwards R. A.. (2008). The RAST server: Rapid annotations using subsystems technology. BMC Genomics 9, 75. doi: 10.1186/1471-2164-9-75 18261238 PMC2265698

[B6] Backer R. Rokem J. S. Ilangumaran G. Lamont J. Praslickova D. Ricci E.. (2018). Plant growth-promoting rhizobacteria: Context, mechanisms of action, and roadmap to commercialization of biostimulants for sustainable agriculture. Front. Plant Sci. 9. doi: 10.3389/fpls.2018.01473 PMC6206271 30405652

[B7] Beneduzi A. Ambrosini A. Passaglia L. M. P. (2012). Plant growth-promoting rhizobacteria (PGPR): Their potential as antagonists and biocontrol agents. Genet. Mol. Biol. 35, 1044–1051. doi: 10.1590/s1415-47572012000600020 23411488 PMC3571425

[B8] Braud A. Jézéquel K. Léger M.-A. Lebeau T. (2006). Siderophore production by using free and immobilized cells of two pseudomonads cultivated in a medium enriched with fe and/or toxic metals (Cr, Hg, Pb). Biotechnol. Bioeng. 94, 1080–1088. doi: 10.1002/bit.20937 16586510

[B9] Bray R. H. Kurtz L. T. (1945). Determination of total, organic, and available forms of phosphorus in soils. Soil Sci. 59, 39–46. doi: 10.1097/00010694-194501000-00006

[B10] Bruto M. Prigent-Combaret C. Muller D. Moënne-Loccoz Y. (2014). Analysis of genes contributing to plant-beneficial functions in plant growth-promoting rhizobacteria and related proteobacteria. Sci. Rep. 4, 6261. doi: 10.1038/srep06261 25179219 PMC4151105

[B11] Cárdenas Espinosa M. J. Schmidgall T. Wagner G. Kappelmeyer U. Schreiber S. Heipieper H. J.. (2021). An optimized method for RNA extraction from the polyurethane oligomer degrading strain pseudomonas capeferrum TDA1 growing on aromatic substrates such as phenol and 2,4-diaminotoluene. PloS One 16, e0260002. doi: 10.1371/journal.pone.0260002 34780548 PMC8592408

[B12] Chiaromonte F. Yap V. B. Miller W. (2002). Scoring pairwise genomic sequence alignments. Pac. Symp. Biocomput., 115–126. doi: 10.1142/9789812799623_0012 11928468

[B13] Choudhary D. K. Sharma K. P. Gaur R. K. (2011). Biotechnological perspectives of microbes in agro-ecosystems. Biotechnol. Lett. 33, 1905–1910. doi: 10.1007/s10529-011-0662-0 21660571

[B14] Conesa A. Gotz S. Garcia-Gomez J. M. Terol J. Talon M. Robles M. (2005). Blast2GO: A universal tool for annotation, visualization and analysis in functional genomics research. Bioinformatics 21, 3674–3676. doi: 10.1093/bioinformatics/bti610 16081474

[B15] Dahmani M. A. Desrut A. Moumen B. Verdon J. Mermouri L. Kacem M.. (2020). Unearthing the plant growth-promoting traits of bacillus megaterium RmBm31, an endophytic bacterium isolated from root nodules of retama monosperma. Front. Plant Sci. 11. doi: 10.3389/fpls.2020.00124 PMC7055178 32174934

[B16] Dhaked B. S. Triveni S. Reddy R. S. Padmaja G. (2017). Isolation and screening of potassium and zinc solubilizing bacteria from different rhizosphere soil. Int. J. Curr. Microbiol. App. Sci. 6, 1271–1281. doi: 10.20546/ijcmas.2017.608.154

[B17] Dworkin M. Foster J. W. (1958). Experiments with some microorganisms which utilize ethane and hydrogen. J. Bacteriol. 75, 592–603. doi: 10.1128/jb.75.5.592-603.1958 13538930 PMC290115

[B18] Eida A. A. Bougouffa S. L’Haridon F. Alam I. Weisskopf L. Bajic V. B.. (2020). Genome insights of the plant-growth promoting bacterium cronobacter muytjensii JZ38 with volatile-mediated antagonistic activity against phytophthora infestans. Front. Microbiol. 11. doi: 10.3389/fmicb.2020.00369 PMC7078163 32218777

[B19] García-Fraile P. Menéndez E. Rivas R. (2015). Role of bacterial biofertilizers in agriculture and forestry. AIMSBOA 2, 183–205. doi: 10.3934/bioeng.2015.3.183

[B20] Getahun A. Muleta D. Assefa F. Kiros S. (2020). Plant growth-promoting rhizobacteria isolated from degraded habitat enhance drought tolerance of acacia (Acacia abyssinica hochst. ex benth.) seedlings. Int. J. Microbiol. 2020, 8897998. doi: 10.1155/2020/8897998 33178283 PMC7646561

[B21] Glick B. (2018). Soil microbes and sustainable agriculture. Pedosphere 28, 167–169. doi: 10.1016/S1002-0160(18)60020-7

[B22] Gouda S. Kerry R. G. Das G. Paramithiotis S. Shin H.-S. Patra J. K. (2018). Revitalization of plant growth promoting rhizobacteria for sustainable development in agriculture. Microbiol. Res. 206, 131–140. doi: 10.1016/j.micres.2017.08.016 29146250

[B23] Grover M. Bodhankar S. Sharma A. Sharma P. Singh J. Nain L. (2021). PGPR mediated alterations in root traits: Way toward sustainable crop production. Front. Sustain. Food Syst. 4. doi: 10.3389/fsufs.2020.618230

[B24] Gupta A. Gopal M. Thomas G. Manikandan V. Gajewski J. Thomas G.. (2014). Whole genome sequencing and analysis of plant growth promoting bacteria isolated from the rhizosphere of plantation crops coconut, cocoa and arecanut. PloS One 9, e104259. doi: 10.1371/journal.pone.0104259 25162593 PMC4146471

[B25] Hudek L. Premachandra D. Webster W. A. J. Bräu L. (2016). Role of phosphate transport system component PstB1 in phosphate internalization by nostoc punctiforme. Appl. Environ. Microbiol. 82, 6344–6356. doi: 10.1128/AEM.01336-16 27542935 PMC5066351

[B26] Jian C. Luukkonen P. Yki-Järvinen H. Salonen A. Korpela K. (2020). Quantitative PCR provides a simple and accessible method for quantitative microbiota profiling. PloS One 15, e0227285. doi: 10.1371/journal.pone.0227285 31940382 PMC6961887

[B27] Kazan K. (2013). Auxin and the integration of environmental signals into plant root development. Ann. Bot. 112, 1655–1665. doi: 10.1093/aob/mct229 24136877 PMC3838554

[B28] Khan M. A. Asaf S. Khan A. L. Jan R. Kang S.-M. Kim K.-M.. (2020). Thermotolerance effect of plant growth-promoting bacillus cereus SA1 on soybean during heat stress. BMC Microbiol. 20, 175. doi: 10.1186/s12866-020-01822-7 32571217 PMC7310250

[B29] Khan N. Bano A. Rahman M. A. Guo J. Kang Z. Babar M. A. (2019). Comparative physiological and metabolic analysis reveals a complex mechanism involved in drought tolerance in chickpea (Cicer arietinum l.) induced by PGPR and PGRs. Sci. Rep. 9, 2097. doi: 10.1038/s41598-019-38702-8 30765803 PMC6376124

[B30] Kishor PB K. (2019). Bacterial cold shock proteins - the molecular chaperones for multiple stress tolerance. AIBM 12, 57–61. doi: 10.19080/AIBM.2019.12.555837

[B31] Kramer J. Özkaya Ö. Kümmerli R. (2020). Bacterial siderophores in community and host interactions. Nat. Rev. Microbiol. 18, 152–163. doi: 10.1038/s41579-019-0284-4 31748738 PMC7116523

[B32] Lalaouna D. Baude J. Wu Z. Tomasini A. Chicher J. Marzi S.. (2019). RsaC sRNA modulates the oxidative stress response of staphylococcus aureus during manganese starvation. Nucleic Acids Res. 47, 9871–9887. doi: 10.1093/nar/gkz728 31504767 PMC6765141

[B33] Larkin M. A. Blackshields G. Brown N. P. Chenna R. McGettigan P. A. McWilliam H.. (2007). Clustal W and clustal X version 2.0. Bioinformatics 23, 2947–2948. doi: 10.1093/bioinformatics/btm404 17846036

[B34] Lastochkina O. Pusenkova L. Yuldashev R. Babaev M. Garipova S. Blagova D.. (2017). Effects of bacillus subtilis on some physiological and biochemical parameters of triticum aestivum l. (wheat) under salinity. Plant Plant Physiol. Biochem. 121, 80–88. doi: 10.1016/j.plaphy.2017.10.020 29096176

[B35] Livak K. J. Schmittgen T. D. (2001). Analysis of relative gene expression data using real-time quantitative PCR and the 2–ΔΔCT method. Methods 25, 402–408. doi: 10.1006/meth.2001.1262 11846609

[B36] Loper J. (1986). Influence of bacterial sources of Indole3-acetic acid on root elongation of sugar beet. Phytopathology 76, 386–389. doi: 10.1094/Phyto-76-386

[B37] Mandal L. Kotasthane A. S. (2014). Isolation and assessment of plant growth promoting activity of siderophore producing pseudomonas fluorescens in crops. *Intern* . Jour. Agricul. Environ. Biotech. 7, 63. doi: 10.5958/j.2230-732X.7.1.009

[B38] Mehlich A. (1984). Mehlich 3 soil test extractant: A modification of mehlich 2 extractant. Commun. Soil Sci. Plant Anal. 15, 1409–1416. doi: 10.1080/00103628409367568

[B39] Mellidou I. Karamanoli K. (2022). Unlocking PGPR-mediated abiotic stress tolerance: What lies beneath. Front. Sustain. Food Syst. 6. doi: 10.3389/fsufs.2022.832896

[B40] Nautiyal C. S. (1999). An efficient microbiological growth medium for screening phosphate solubilizing microorganisms. FEMS Microbiol. Lett. 170, 265–270. doi: 10.1111/j.1574-6968.1999.tb13383.x 9919677

[B41] Nelson D. w. Sommers L. e. (1983). “Total carbon, organic carbon, and organic matter,” in Methods of soil analysis (John Wiley & Sons, Ltd), 539–579. doi: 10.2134/agronmonogr9.2.2ed.c29

[B42] Nezarat S. Gholami A. (2009). Plant growth promoting rhizobacteria for improving seed germination, seedling growth and yield of MaizeScreening. Pakistan journal of biological sciences. PJBS 12, 26–32. doi: 10.3923/pjbs.2009.26.32 19579914

[B43] Niu X. Song L. Xiao Y. Ge W. (2018). Drought-tolerant plant growth-promoting rhizobacteria associated with foxtail millet in a semi-arid agroecosystem and their potential in alleviating drought stress. Front. Microbiol. 8. doi: 10.3389/fmicb.2017.02580 PMC5771373 29379471

[B44] Noori F. Etesami H. Noori S. Forouzan E. Salehi Jouzani G. Malboobi M. A. (2021). Whole genome sequence of pantoea agglomerans ANP8, a salinity and drought stress–resistant bacterium isolated from alfalfa (Medicago sativa l.) root nodules. Biotechnol. Rep. 29, e00600. doi: 10.1016/j.btre.2021.e00600 PMC7893418 33643858

[B45] Paul D. Lade H. (2014). Plant-growth-promoting rhizobacteria to improve crop growth in saline soils: A review. Agron. Sustain. Dev. 34, 737–752. doi: 10.1007/s13593-014-0233-6

[B46] Paul D. Sinha S. (2013). Phosphate solubilizing activity of some bacterial strains isolated from jute mill effluent exposed water of river ganga. Indian J. Fundam. Appl. Life Sci. 3, 39–45.

[B47] Ramadoss D. Lakkineni V. K. Bose P. Ali S. Annapurna K. (2013). Mitigation of salt stress in wheat seedlings by halotolerant bacteria isolated from saline habitats. Springerplus 2, 6. doi: 10.1186/2193-1801-2-6 23449812 PMC3579424

[B48] Raza W. Ling N. Yang L. Huang Q. Shen Q. (2016). Response of tomato wilt pathogen ralstonia solanacearum to the volatile organic compounds produced by a biocontrol strain bacillus amyloliquefaciens SQR-9. Sci. Rep. 6, 24856. doi: 10.1038/srep24856 27103342 PMC4840334

[B49] Saitou N. Nei M. (1987). The neighbor-joining method: A new method for reconstructing phylogenetic trees. Mol. Biol. Evol. 4, 406–425. doi: 10.1093/oxfordjournals.molbev.a040454 3447015

[B50] Sapre S. Gontia-Mishra I. Tiwari S. (2018). Klebsiella sp. confers enhanced tolerance to salinity and plant growth promotion in oat seedlings (Avena sativa). Microbiol. Res. 206, 25–32. doi: 10.1016/j.micres.2017.09.009 29146257

[B51] Sashidhar B. Podile A. R. (2010). Mineral phosphate solubilization by rhizosphere bacteria and scope for manipulation of the direct oxidation pathway involving glucose dehydrogenase. J. Appl. Microbiol. 109, 1–12. doi: 10.1111/j.1365-2672.2009.04654.x 20070432

[B52] Schwyn B. Neilands J. B. (1987). Universal chemical assay for the detection and determination of siderophores. Anal. Biochem. 160, 47–56. doi: 10.1016/0003-2697(87)90612-9 2952030

[B53] Shen X. Hu H. Peng H. Wang W. Zhang X. (2013). Comparative genomic analysis of four representative plant growth-promoting rhizobacteria in pseudomonas. BMC Genomics 14, 271. doi: 10.1186/1471-2164-14-271 23607266 PMC3644233

[B54] Suarez C. Ratering S. Hain T. Fritzenwanker M. Goesmann A. Blom J.. (2019). Complete genome sequence of the plant growth-promoting bacterium hartmannibacter diazotrophicus strain E19T. Int. J. Genomics 2019, 7586430. doi: 10.1155/2019/7586430 31583244 PMC6754898

[B55] Subramanian P. Kim K. Krishnamoorthy R. Mageswari A. Selvakumar G. Sa T. (2016). Cold stress tolerance in psychrotolerant soil bacteria and their conferred chilling resistance in tomato (Solanum lycopersicum mill.) under low temperatures. PloS One 11, e0161592. doi: 10.1371/journal.pone.0161592 27580055 PMC5006972

[B56] Sukumar P. Legué V. Vayssières A. Martin F. Tuskan G. A. Kalluri U. C. (2013). Involvement of auxin pathways in modulating root architecture during beneficial plant–microorganism interactions. Plant. Cell Environ. 36, 909–919. doi: 10.1111/pce.12036 23145472

[B57] Tamura K. Stecher G. Kumar S. (2021). MEGA11: Molecular evolutionary genetics analysis version 11. Mol. Biol. Evol. 38, 3022–3027. doi: 10.1093/molbev/msab120 33892491 PMC8233496

[B58] Timmusk S. Behers L. Muthoni J. Muraya A. Aronsson A.-C. (2017). Perspectives and challenges of microbial application for crop improvement. Front. Plant Sci. 8. doi: 10.3389/fpls.2017.00049 PMC5299024 28232839

[B59] Vasseur-Coronado M. du Boulois H. D. Pertot I. Puopolo G. (2021). Selection of plant growth promoting rhizobacteria sharing suitable features to be commercially developed as biostimulant products. Microbiol. Res. 245, 126672. doi: 10.1016/j.micres.2020.126672 33418398

[B60] Vincent J. M. (1970). “A manual for the practical study of the root-nodule bacteria,” in A manual for the practical study of the root-nodule bacteria. IBP Handbk 15 Oxford and Edinburgh: Blackwell Scientific Publications. Available at: https://www.cabdirect.org/cabdirect/abstract/19710700726.

[B61] Vurukonda S. S. K. P. Vardharajula S. Shrivastava M. SkZ A. (2016). Enhancement of drought stress tolerance in crops by plant growth promoting rhizobacteria. Microbiol. Res. 184, 13–24. doi: 10.1016/j.micres.2015.12.003 26856449

[B62] Wagh J. Shah S. Bhandari P. Archana G. Kumar G. N. (2014). Heterologous expression of pyrroloquinoline quinone (pqq) gene cluster confers mineral phosphate solubilization ability to herbaspirillum seropedicae Z67. Appl. Microbiol. Biotechnol. 98, 5117. doi: 10.1007/s00253-014-5610-1 24682480

[B63] Weisburg W. G. Barns S. M. Pelletier D. A. Lane D. J. (1991). 16S ribosomal DNA amplification for phylogenetic study. J. Bacteriol. 173, 697–703. doi: 10.1128/jb.173.2.697-703.1991 1987160 PMC207061

[B64] Zaid D. S. Cai S. Hu C. Li Z. Li Y. (2022). Comparative genome analysis reveals phylogenetic identity of bacillus velezensis HNA3 and genomic insights into its plant growth promotion and biocontrol effects. Microbiol. Spectr. 10, e02169–e02121. doi: 10.1128/spectrum.02169-21 35107331 PMC8809340

