# Supplementary figures and images for: Temperature-induced modulation of stress-tolerant PGP genes bioprospected from Bacillus sp. IHBT-705 associated with saffron (Crocus sativus) rhizosphere: A natural -treasure trove of microbial biostimulants
Source: Front Plant Sci. 2023 Feb 27;14:1141538. doi: 10.3389/fpls.2023.1141538 (PMC10009223; doi:10.3389/fpls.2023.1141538)

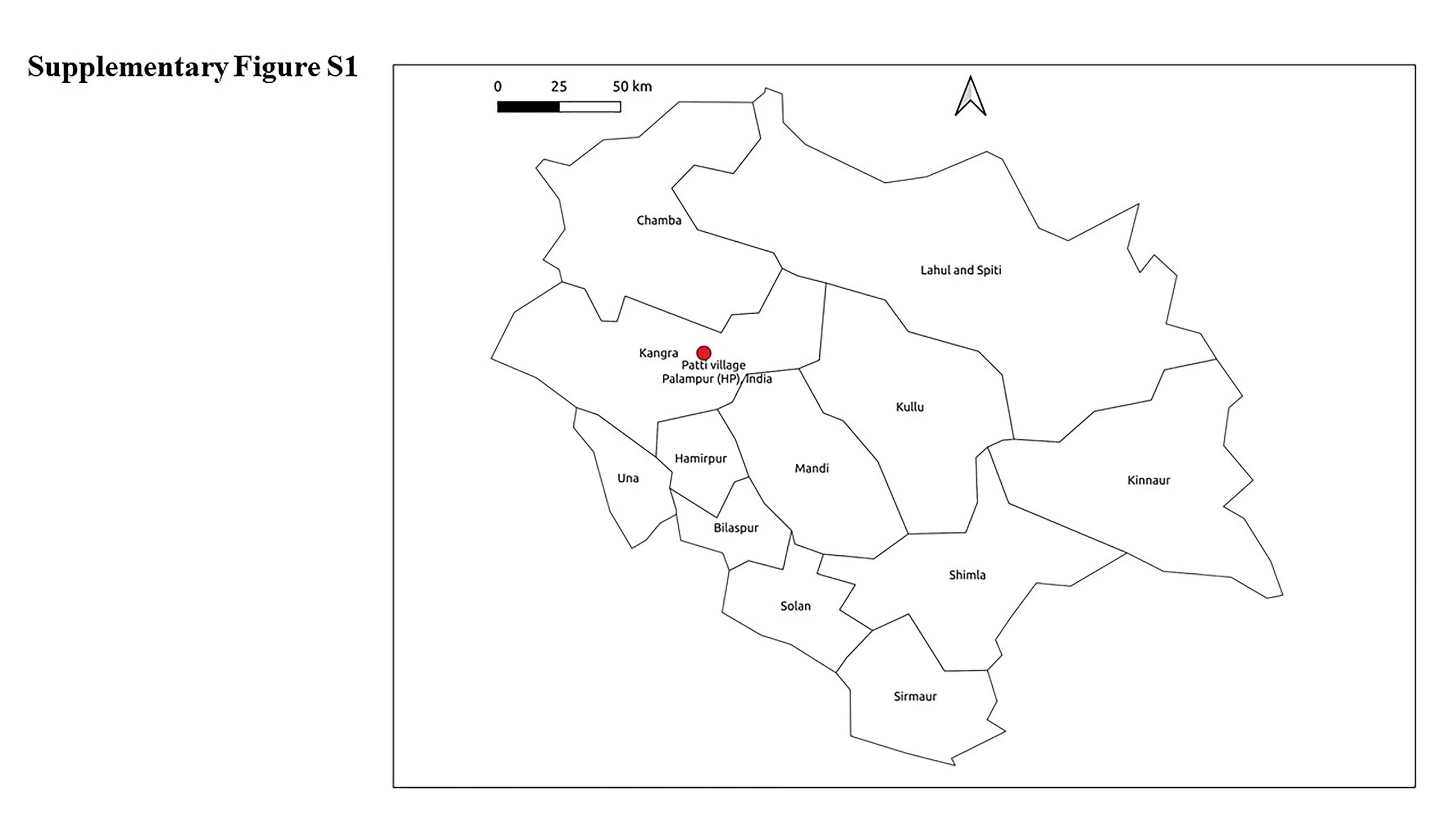

Supplement: Supplementary Figure 1 — Map of soil sampling site for isolation of PGPR. [file Image_1.jpeg]

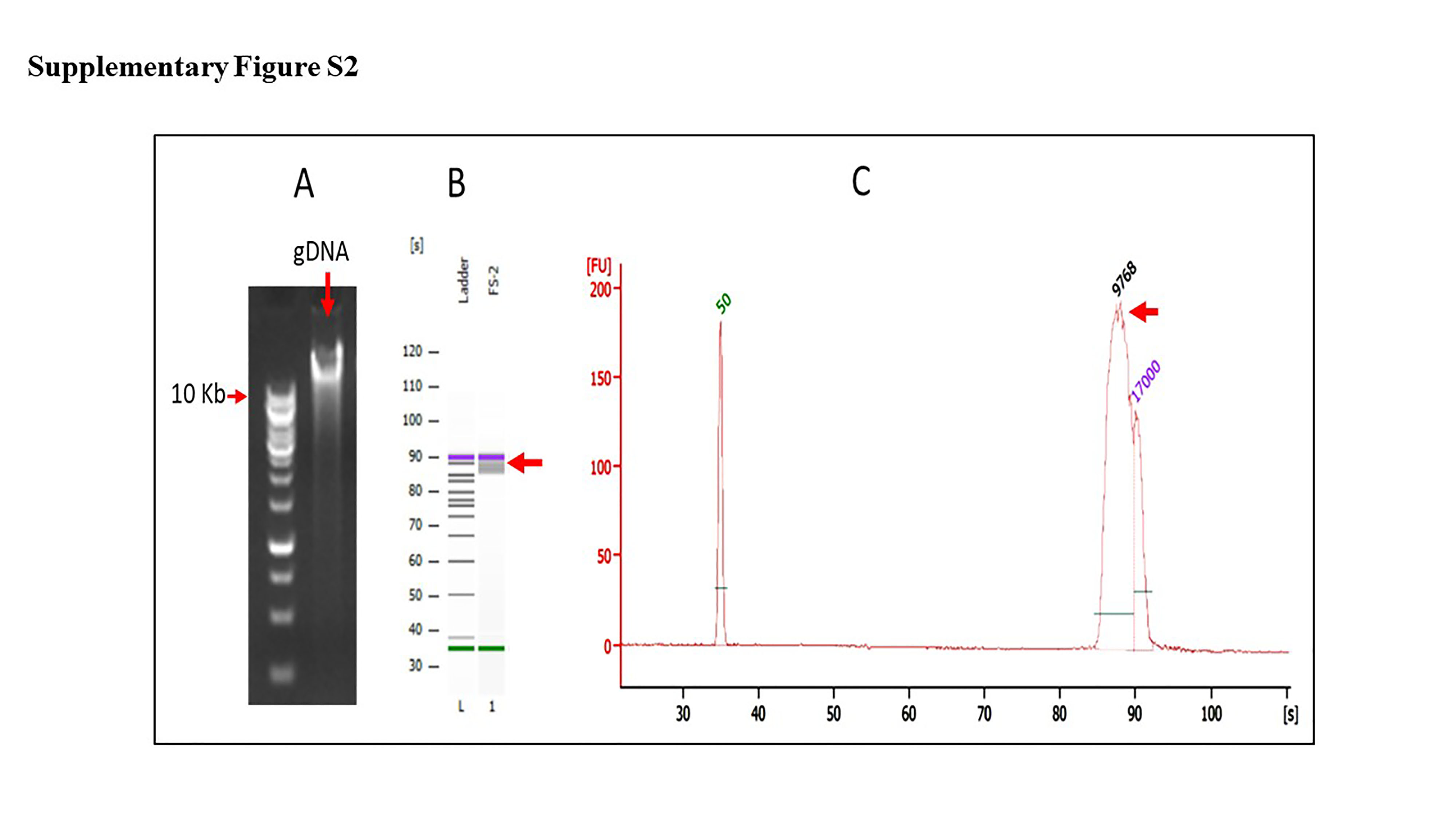

Supplement: Supplementary Figure 2 — Library validation of gDNA for whole genome sequencing of Bacillus sp. IHBT-705. (A) Gel profile of isolated high-quality gDNA of Bacillus sp. IHBT-705 with 1Kb ladder. (B) Bioanalyzer profile of Bacillus sp. IHBT-705 SMRTbell templates for sequencing with ladder. (C) Bioanalyzer profile of prepared library size (~9.8 Kb) of Bacillus sp. IHBT-705. [file Image_2.jpeg]

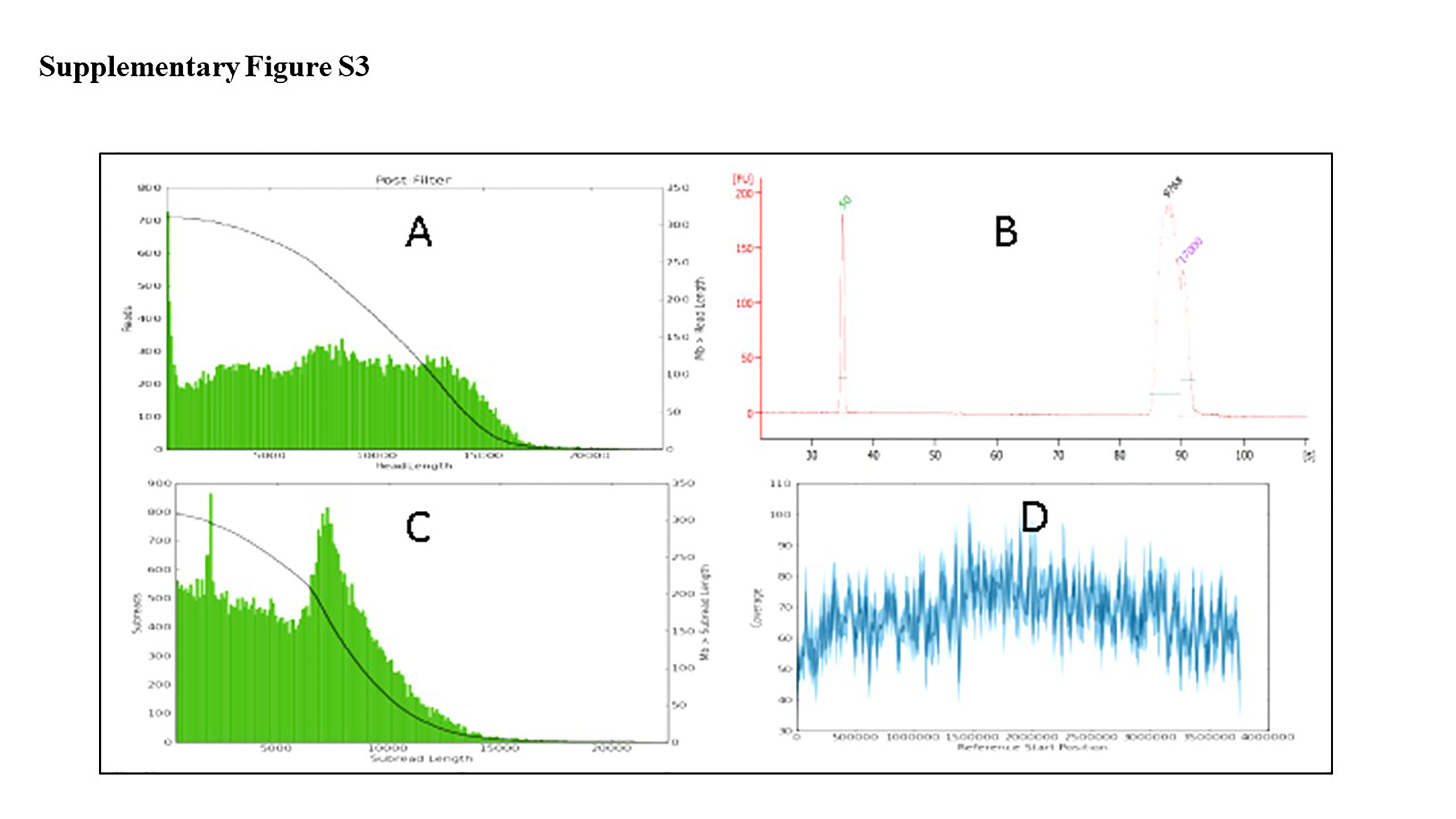

Supplement: Supplementary Figure 3 — Genome assembly features of PacBio RS II data using HGAP v3.0. (A) Polymerase read length distribution of post-filter reads. (B) Polymerase read quality distribution of post-filter reads. (C) Subread filtering of the whole genome of Bacillus sp. IHBT-705 sequencing reads. (D) Coverage across a reference of single contig assembly (with an average coverage of 69X). [file Image_3.jpeg]

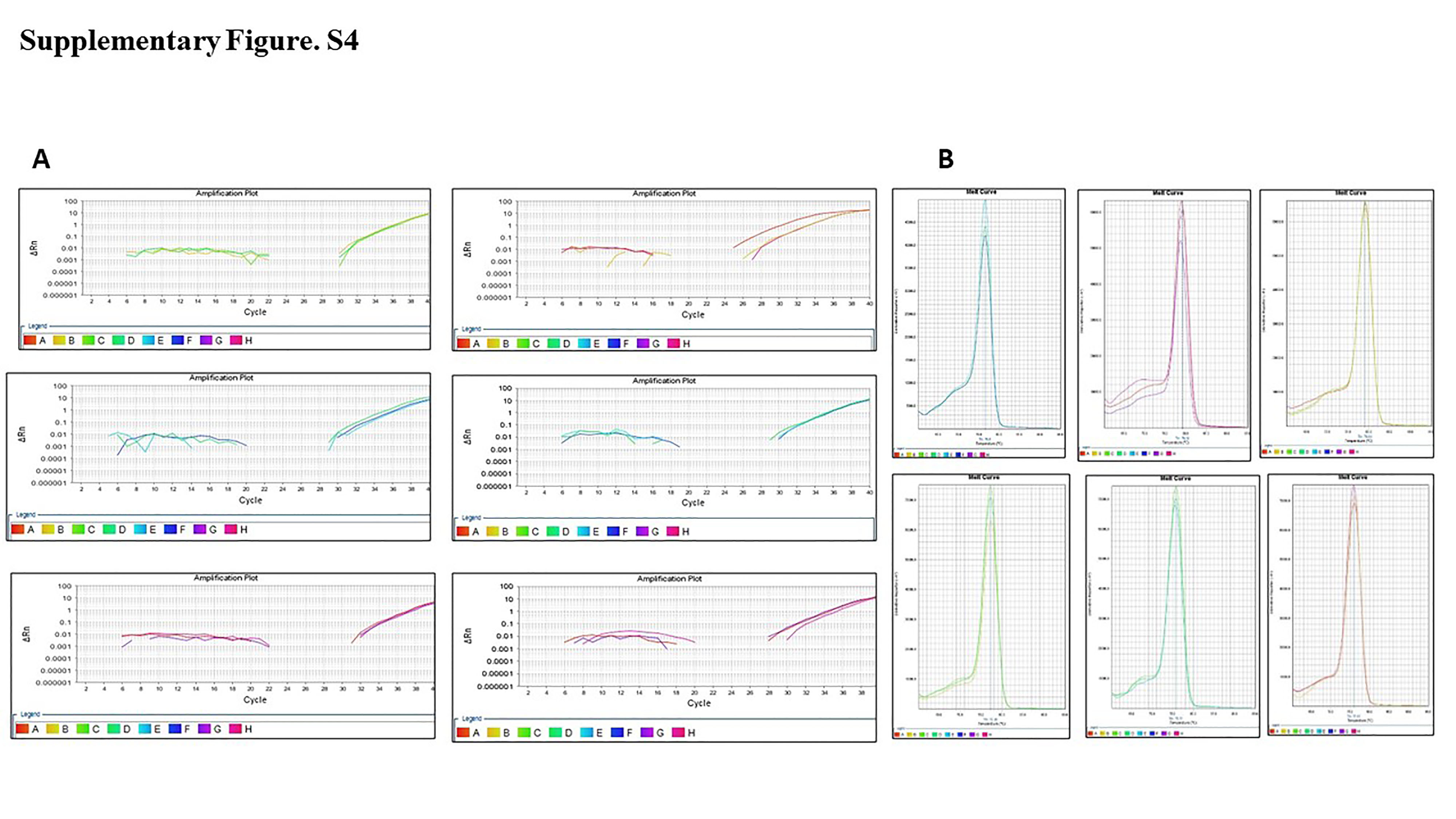

Supplement: Supplementary Figure 4 — Expressions analysis of targeted stress tolerant and PGP genes by qRT-PCR. (A) Amplification plots (B) Melt curves of targeted genes. [file Image_4.jpeg]
