# Supplementary material for: Temperature-induced modulation of stress-tolerant PGP genes bioprospected from Bacillus sp. IHBT-705 associated with saffron (Crocus sativus) rhizosphere: A natural -treasure trove of microbial biostimulants
Source: Front Plant Sci. 2023 Feb 27;14:1141538. doi: 10.3389/fpls.2023.1141538 (PMC10009223; doi:10.3389/fpls.2023.1141538)
Supplement: Supplementary file 5 [file Table_1.docx]

**Supplementary Table S1** Targeted gene-specific primer sequences of stress-tolerant and PGP genes used in RT-qPCR for expression analysis

| **S. No** | **Gene** | **Primer Sequence 5'--> 3'** |
| --- | --- | --- |
| 1 | *nhaX* | Forward ATGACTGGCGGCGCTTATAT  Reverse CAAGGGACTTTGCTGTTCATCA |
| 2 | *cspD* | Forward CGAAGGTGACGGTTACAAATCTC  Reverse CACGATTACCTTCAACGATTTCAA |
| 3 | *cspB* | Forward CAAGGCGAAGGCTTCAAAAC  Reverse GCTTGTGGTCCACGGTTACC |
| 4 | *cspC* | Forward CAGCTATCCAAGGTGACGGATT  Reverse CAGCTTGAGCTCCACGAGAAC |
| 5 | *dnaJ* | Forward ATGTGAGGGCGCATGAATTC  Reverse GCAAAGGTAAGCGGCATTTC |
| 6 | *dnaK* | Forward CAGCAGCTGCACTTGCTTATG  Reverse TGTACCGCCACCAAGGTCAT |
| 7 | *hslO* | Forward AATCAACGAAGCGCAGAGAAG  Reverse TGTCATGGTTCTGCCGATTG |
| 8 | *htpX* | Forward AGAAGATCGCTTGTTGCCGTAT  Reverse TAACCCCTTCAACTGCTGCAT |
| 9 | *grpE* | Forward GAAGGCGTTGAACCAATTGAA  Reverse CTTGCATGACGGCTTGATGA |
| 10 | *trpA* | Forward CACCAACAAGCAAGGAACGA  Reverse GACGCCGAGAGATGAAACACA |
| 11 | *trpB* | Forward TCTCGGCGGTGCTAAAATCT  Reverse GCCCAAGCGCATTGTTAATT |
| 12 | *trpD* | Forward GCGGCTCGTTAACAGATGCT  Reverse TCACGAAGCCTGTCATTTCTTC |
| 13 | *trpC* | Forward AAACAGCACGGAGCAAACG  Reverse CGGCTTTCTCCTGTGATCGT |
| 14 | *trpE* | Forward CGACGGCAATATCGATTCCT  Reverse CGGCACCTGCTTGTATTGAA |
| 15 | *fhuC* | Forward TTTACCGCAGACGCCTGAAG  Reverse CGGCCAAATCCATTTTGATG |
| 16 | *fhuD* | Forward TGCCTCAAAACCCACAACGT  Reverse TTGCCGAGTGTGACGAGATC |
| 17 | *fhuB* | Forward ACGGTATGGGAAGCCGTCTT  Reverse GTACGCGGCAGCCTTAGTTC |
| 18 | *fhuG* | Forward TGGCGCTTCTATCGCTGTATC  Reverse GCCGGGATCAGCTAAACCAT |
| 19 | *acrb5* | Forward CGGAGCGAGCCTGATCAC  Reverse AGCCCATCCTCTGCCATTC |
| 20 | *rimM* | Forward CACGAAATCATCGGCTGTGA  Reverse GCCCCTGGCGTTAAAACTTC |
| 21 | *nhaK* | Forward CGGAAGAAATTGGCGTATCC  Reverse TCTTGCTCGATGGCATGTGT |
| 22 | *pstB1* | Forward GCAGCAGCGTCTTTGTATCG  Reverse CAAGCGCAGATGTTGGTTCA |
| 23 | *xerC1* | Forward GATGGAAGGCAGAAGCTGAAA  Reverse CGCGCTGATTCAAAAACACA |
| 24 | *xerC2* | Forward GAAGAGGGTATGGCAAGAGCAA  Reverse TTTAGCCGCTTCTGAATGTTGTT |
| 25 | Reference *16S* | Forward AGACACGGCCCAGACTCCTA  Reverse CTCCGTCAGACTTTCGTCCATT |
